# Buffalo Medical and Surgical Association

**Published:** 1884-12

**Authors:** Frederick Peterson

**Affiliations:** Secretary


### Society Reports.

### Buffalo Medical and Surgical Association.

*Regular Meeting Oct. 7, 1884.*

President, F. W. Bartlett, in the Chair.

The minutes of the last meeting were read and approved.

Drs. Niemand and Bode applied for membership.

Drs. Grosvenor and Appel were invited to participate in the proceedings.

Dr. Roswell Park exhibited some interesting specimens of tubecular disease of bone, and spoke at some length upon the frequency of such disorders. He was inclined to believe, with the latest German and French writers upon the subject, that most cases of caries, hip-joint disease, etc., had their common origin in the infectious contagium of tubercle. There was a peculiar aversion among American and English surgeons to the acceptance of this theory.

In the discussion which followed, Dr. Ring led. He referred to Dr. Henry Smith's lectures in Philadelphia upon tuberculous disease of bone.

Dr. Van Peyma asked why Dr. Park thought these specimens which he exhibited of a tubercular nature.

Dr. Wetmore disagreed with the speaker in some points he made. He believed the majority of cases of hip-joint disease originated from traumatic and not tubercular causes. A concussion produced an ecchymosis in the basement membrane of the joint. This, irritated by walking and other motion, finally resulted in ulceration. If there was anything tubercular in the disease in the larger number of cases, it would return, but experience had not borne this out.

Dr. Hartwig was inclined to accept Dr. Park's views. Although as a rule we can ascertain that a fall has been the commencement of the hip-joint disorder, tubercle is not thus excluded. He mentioned a case in illustration, where he had operated upon a knee three times, the last an amputation because the disease returned each time, and even after amputation; he is fearful of his not continuing in good health. Upon the whole, he thought the opinions of Drs. Park and Wetmore might be reconciled.

Dr. Park, in answer to Dr. Van Peyma, stated that he had found bacilli in his specimens, and also the histological structure of tubercle. He spoke of the value of ignipuncture in the treatment of these cases.

Dr. Phelps had recently operated upon two cases of white swelling of the knee. One was a little girl, the other a man of twenty-seven. There was a history of tubercle in both, but only one began with a decided traumatic cause. He had no doubt there was tubercular disease of the lower part of the femur in both.

Dr. Peterson made a report of the pathological results of two hundred and fifteen post mortem examinations. He said that he made this report more particularly to show what a student of pathology and pathological anatomy and histology might obtain in Buffalo in the way of material; that the opportunities were, in fact, better than those in New York or other large cities, where the material was divided among many. Of these 215 cases, 204 died from disease or violent death, 11 from suicide. These autopsies were upon cases at the Buffalo General Hospital, Erie County Almshouse, State Insane Asylum, and private and coroners' cases.

The ages were from birth to 89 years, and are tabulated as follows:

Under 1 year, 7 cases; between 1 and 10 years, 5 cases; 10 and 20 years, 11 cases; 20 and 30 years, 43 cases; 30 and 40 years, 39 cases; 40 and 50 years, 50 cases; 50 and 60 years, 26 cases; 60 and 70 years, 16 cases; 70 and 80 years, 14 cases; 80 and 90 years, 4 cases.

The causes of death, where known, were written in the death certificates as follows:

Tuberculosis, 27 cases; cardiac disease, 26 cases; violent deaths (exclusive of drowning), 21 cases; pneumonia, 18 cases; drowning, 15 cases; malignant tumors, 14 cases; septic diseases, 11 cases; renal diseases, 9 cases; diseases of alimentary canal, 8 cases; cerebral hemorrhage, 7 cases; senile involution, 7 cases; peritonitis, 6 cases; cirrhosis of liver, 6 cases; acute infectious fevers, 6 cases; pachymeningitis, 5 cases; alcoholism, 4 cases; convulsions, 4 cases; gangrene of lungs, 4 cases; aneurism, 3 cases; abdominal hemorrhage, 2 cases; acute yellow atrophy of liver, 1 case; air in the veins, 1 case; purpura hemorrhagica, 1 case; kyphosis, one case.

But as in each carefully conducted autopsy from two to twenty pathological conditions are always found, the speaker had tabulated, in the following manner, the morbid states he had seen and examined:

### TUBERCULOSIS.

There were 32 cases in which there was tuberculosis of lungs; 14 of kidneys; 13 of ileum; 7 of liver; 7 of spleen; 7 of meninges; 5 of peritoneum; 3 of cœcum; 3 of larynx; 2 of pleuræ; 1 of brain; 1 of prostate gland; 1 of pelvis of kidney; 1 of medulla.

### TUMORS.

In 3 cases there was primary carcinoma of stomach; 1 of liver; 1 of œsophagus; 1 of gall-bladder; 1 of rectum; 1 of pancreas. In 3 cases there was secondary carcinoma of liver; 3 of mesenteric glands; 2 of lungs; 1 of glands of neck; 1 of heart; 1 of peritoneum; 1 of bronchial glands; 1 of ovary; 1 of alveolar sarcoma of pleuræ and peritoneum; 2 of round-celled sarcoma of neck; 1 of medullary sarcoma of pituitary body; 1 of medullary sarcoma of cerebellum; 1 of medullary sarcoma of brain; 1 of medullary sarcoma of meninges; 5 of myofibroma of uterus; 2 of fibroma of liver; 1 of fibroma of kidney; 1 of fibroma of mitral valve; 1 of lymphoma of neck; 1 of adenoma of stomach; 2 of epithelioma of nose; 1 of papilloma of bladder; 1 of atheroma of skin over sternum; 1 of atheroma of skin upon penis; 1 of tumor cavernosus of liver; 1 of psammoma of dura mater; 7 of ovarian cysts.

### MALFORMATIONS.

In three cases there was congenital hour-glass contraction of stomach; 1 case of corset displacement of stomach; 5 of corset atrophy of liver; 3 of persisting fœtal lobulation of kidneys; 1 of floating kidney; 1 of cryptorshismus or hermaphrotismus; 1 of double kidney on one side; 1 of diverticulum of the ileum; 2 of diverticula of the bladder.

### PARASITES.

In two cases were found echinococci in the liver, and in numerous cases tæniæ, ascarides and trichocephalus diapar.

### FATTY DEGENERATION AND INFILTRATION.

As in most acute diseases, septic or inflammatory, and in many chronic diseases, like tuberculosis, there is great interference with cellular nutrition, these fatty changes are very common. Hence, in these 215 post mortem examinations, one need not be surprised to find that there were 72 cases of fatty metamorphosis of the heart, 72 of the liver, 46 of the kidneys, 3 of recti abdominis.

### DISEASES OF SEROUS MEMBRANES.

Brain—In 20 cases there was chronic hydrocephalus internus; 5 of chronic meningitis; 5 of meningeal hemorrhages; 4 of chronic pachymeningitis; 2 of acute hydrocephalus internus; 1 of suppurative pachymeningitis.

Pleuræ—In 19 cases there were no adhesions of pleuræ; in 196 cases there were old adhesions of pleuræ; in 31 cases there was acute pleuritis; 17 cases of hydrothorax; 9 of "spots of Tardieu" on pleuræ; 6 of empyema; 3 of hæmothorax; 3 of pneumothorax; in 2 there were lime plates in pleuræ.

Pericardium—In 37 cases there was pericardial dropsy. The speaker had found that in all cases where there is acute or chronic disease of the lungs, there is a great increase of serum in the pericardium. In 31 cases there were "soldier spots" on pericardium; 10 cases of old adhesions of pericardium; 10 of "spots of Tardieu" on pericardium; 5 of acute pericarditis; 1 of œdema of pericardium.

Peritoneum—In 23 cases there was acute peritonitis; 13 cases of peritoneal dropsy; 10 of chronic peritonitis; 6 of chronic pelvic peritonitis; 3 of "spots of Tardieu"; 1 of hemorrhage into cavity; 1 of encysted dropsy.

The diseases of the various organs, separately examined, were as follows:

### BRAIN.

In 5 cases there was atrophy of brain; 4 of softening of brain; 3 of congestion of brain; 3 of cysts in choroid plexus; 3 of clots in cerebrum; 3 of clots in pons varolii; 3 of clots in optic thalamus; 2 of embolism of middle cerebral artery; 1 of multiple clots in cerebrum; 1 of abscess of brain; 1 of cyst of pineal gland; 1 of œdema of brain.

### LUNGS.

In 34 cases there was emphysema; 24 cases of croupous pneumonia; 18 of bronchitis; 13 of catarrhal pneumonia; 8 of œdema; 6 of old healed tubercles; 5 of gangrene; 4 of infarctions; 3 of abscess; 3 of chronic pneumonia; 2 of schluchpneumonie.

### HEART.

In 69 cases there were atheromatous arteries, in which change, as a rule, does not manifest itself until after the fortieth year. But in three of these cases the ages were 22, 27, 27 years, respectively.

In 17 cases there was aortic stenosis; 17 cases of thickened mitral; 16 of agonia clots; 14 of vegetations on mitral; 12 of hypertrophy of heart; 11 of atrophy of heart; 11 of thickened aortic; 8 of mitral stenosis; 7 of mitral insufficiency; 7 of aortic insufficiency; 4 of brown atrophy; 4 of dilatation; 3 of tricuspid insufficiency; 3 of vegetations on aortic; 2 of rupture of heart; 1 of penetration of aortic; 1 of thickened tricuspid; 1 of thickened pulmonary; 1 of thrombus cardis (both sides); 1 of cor adiposum.

### SPLEEN.

In 33 cases there was acute swelling; 14 cases of chronic swelling; 8 of infarction; 8 of calcified capsule; 7 of atrophy; 2 of amyloid degeneration; 2 of cyanotic induration.

### LIVER.

In 23 cases there was cirrhosis; 15 cases of atrophy; 8 of gall-stones; 4 of hypertrophy; 3 of red atrophy; 3 of cyanotic induration; 3 of syphilitic hepatitis; 3 of acute hepatitis; 2 of acute yellow atrophy; 2 of amyloid degeneration; 1 of chronic hepatitis; 1 of rupture of liver; 1 of catarrh of gall-bladder.

### GENITO-URINARY ORGANS.

In 30 cases there was chronic interstitial nephritis; 11 cases of chronic atrophy; 9 of acute cystitis; 8 of pyelonephritis; 7 of acute parenchymatous nephritis; 7 of infarction of kidneys; 6 of pyelitis; 5 of cyanotic induration; 4 of colloid cysts of large size in kidney; 4 of enlarged prostate; 3 of chronic cystitis; 2 of hydronephrosis; 2 of amyloid degeneration; 2 of perinephritis; 2 of ureteritis; 2 of stricture of male urethra; 2 of dilated ureters by pregnancy; 1 of gumma of kidney; 1 of pyonephrosis; 1 of calculi in urine-tubules; 1 of hydrocele. In 1 case there was a calculus impacted in each ureter near the pelvis of the kidney, at the same time, causing acute suppression of urine and death.

### ALIMENTARY CANAL.

In 32 cases there was chronic gastric catarrh; 9 cases of acute gastric catarrh; 8 of enlarged mesenteric glands; 5 of catarrh of colon; 4 of catarrh of ileum; 4 of typhoid ulcers in colon; 4 of typhoid ulcers in ileum; 3 of contractions of colon; 3 of chronic enteritis; 2 of chronic perforating ulcers of stomach; 2 of perforation of ileum; 2 of perforation of colon; 2 of ulcers in rectum; 2 of catarrh of rectum; 2 of ulcers in duodenum; 2 of duodenitis; 1 of abnormally thin-walled stomach; 1 of chronic perforating ulcer of duodenum; 1 of amyloid degeneration of intestines; 1 of typhoid ulcers in cœcum; 1 of perforation of cœcum; 1 of typhlitis; 1 of swelling of Peyer's plaques; 1 of stricture of rectum; 1 of calculi in appendix vermiformis; 1 of new-formed passage between hepatic flexure of colon and cœcum, thus cutting off several inches of the ascending colon. This was due to ulceration and peritonitis. In 8 cases there were hernias, scrotal, inguinal, femoral, internal and umbilical.

### FEMALE GENITAL ORGANS.

In 7 cases the uterus was displaced and adhered; 5 cases of acute metritis; 4 of adhered ovary; 3 of hæmatoma of ovary; 3 of hydrosalpinx; 2 of vaginitis; 2 of endometritis; 1 of vagino-recto-perineal fistula. He had noticed that the prostitutes he had examined had morbid conditions of their wombs or ovaries.

### BONE.

In 11 cases there was necrosis, and this was of the nasal, spinal, and bones of leg, arm and skull.

There were two psoas abscesses.

There were three cases of acute general cellulitis or phlegmonous erysipelas.

He had, in these 215 cases, looked attentively at the lungs with reference to the amount of carbonaceous matter in them. Country people and sailors seemed to have little, but city people, by breathing dusty and smoky atmospheres, accumulated, as they progressed in years, large quantities of carbonaceous matter in their lungs. In children the lungs were of a pinkish or pale color. As they grew older, the lungs became more and more discolored. In the course of his remarks he mentioned the fact that by the peculiar method of embalming employed by undertakers in four cases, it had been impossible to recognize pathological changes. One of these was particularly flagrant, as the case was one in charge of the coroner, where there was good reason to suspect poisoning.

Several physicians expressed themselves adverse to these practices of undertakers, among them Drs. Phelps and Cronyn.

*Prevailing Diseases*—Dr. Hartwig reported dysentery and typhoid fever; Dr. Cronyn, whooping-cough and dysentery; Dr. Bartlett, scarlet fever, typhoid fever and dysentery.

Some discussion was then carried on in relation to the publication of the minutes before or after they had been read and approved at the following meeting. Dr. Hartwig and others were in favor of having them published authoritatively, only after approval by the association. The majority of those present concurred in this opinion. Action upon this question was, however deferred until the next session.

The meeting then adjourned.

FREDERICK PETERSON, *Secretary.*

